# Supplementary material for: Potential Benefits of Dietary Plant Compounds on Normal and Tumor Brain Cells in Humans: In Silico and In Vitro Approaches
Source: Int J Mol Sci. 2023 Apr 17;24(8):7404. doi: 10.3390/ijms24087404 (PMC10139435; doi:10.3390/ijms24087404)
Supplement: Supplementary file 1 [file ijms-24-07404-s001.zip › ijms-2311964-supplementary.pdf]

Supplementary Materials

Potential Benefits of Dietary Plant Compounds on Normal and Tumor Brain Cells in Humans: In Silico and In Vitro Approaches

Lucia Camelia Pirvu [1,*], Georgeta Neagu [2], Adrian Albulescu [2,3], Amalia Stefaniu [1] and Lucia Pintilie [4,*]

[1] Department of Pharmaceutical Biotechnologies, National Institute of Chemical Pharmaceutical Research and Development—ICCF, 112 Vitan Av., 031299 Bucharest, Romania
[2] Department of Pharmacology, National Institute of Chemical Pharmaceutical Research and Development—ICCF, 112 Vitan Av., 031299 Bucharest, Romania
[3] Stefan S. Nicolau Institute of Virology, 285 Mihai Bravu Av., 030304 Bucharest, Romania
[4] Department of Synthesis of Bioactive Substances and Pharmaceutical Technologies, National Institute of Chemical Pharmaceutical Research and Development—ICCF, 112 Vitan Av., 031299 Bucharest, Romania
* Correspondence: lucia.pirvu@yahoo.com or lucia.pirvu1@gmail.com (L.C.P.); lpintilie@ncpri.ro (L.P.)

Table S1. The list of the intermolecular interactions between the native ligand (curcumin) and the 20 test vegetal compounds docked with 5ZTN (CLC Software)

Test Compounds	Score	RMSD	Group interaction	Hydrogen bond	Bond Length Å
**Co-crystallized CUR A501 CURCUMIN (native ligand)**	-83.92	1.99	PHE 160, GLY 197, GLU 193, PHE 296, LEU 197, ASP 295, MET 226, LYS 178, VAL 163, ILE 155, ASN 280, ILE 294, ILE 212, LEU 282, PHE 228, ALA 176, ILE 155, LYS 153, LYS 165, **SER** 232, LEU 231, LEU 230, GLU 229, ILE 212, LEU 282, MET 233.	O sp^3 (O1) – N sp^3 from LYS 178 O sp^3 (O3) – N sp^3 from LYS 178<br>O sp^3 (O3) – O sp^2 from GLU 193	3.102<br>2.661<br>3.163
**ANEMONIN**	-34.27	0.003	PHE 160, GLU 193, PHE 296, ASP 295, LYS 178, VAL 163, VAL 164, MET 226, ILE 294, VAL 164, LEU 282, ILE 212, THR 227, ALA 176, PHE 228, LEU 231, GLU 229	-	-
**Apigenin**	-70.83	0.02	GLU 229, LEU 230, LEU 231, PHE 228, **SER** 232, ALA 176, ILE 212, LEU 197, LEU 282, ILE 294, GLU 193, PHE 296, ASP 295, GLY 297, GLY 156, ILE 155, VAL 163, LYS 178.	O sp^2 (O3) – N sp^2 from LEU 231<br>O sp^3 (O2) – N sp^2 from LEU 231<br>O sp^3 (O2) – O sp^2 from LEU 231<br>O sp^3 (O4) – O sp^2 from ILE 155<br>O sp^3 (O5) – O sp^2 from GLU 193	2.897<br>2.741<br>3.098<br>2.989<br>2.736

***o*-Coumaric acid**	-49.70	0.09	LEU 212, LEU 197, GLU 229, LEU 282, LEU 231, LEU 230, **SER** 232, ILE 294, PHE 228, ASP 295, PHE 296, GLU 193, GLU 297, ALA 176, LYS 178, PHE 160, VAL 163, ILE 155.	O sp^3 (O2) – N sp^2 from LEU 231<br>O sp^3 (O2) – O sp^2 from GLU 193	2.987<br>2.577
**Elenoic acid**	-45.42	0.41	PHE 160, GLY 156, ILE 155, VAL 163, LYS 178, LEU 177, ALA 176, MET 226, ASP 295, PHE 296, GLU 193, ILE 294, **SER** 232, LEU 230, LEU 231, GLU 229, ILE 212, PHE 228, LEU 282.	O sp^3 (O4) – O sp^2 from ILE 155<br>O sp^2 (O5) – N sp^2 from LEU 231	2.938<br>2.986
**Hydroxytyrosol**	-50.26	0.17	LEU 282, VAL 293, ILE 294, ASP 295, PHE 296, GLY 297, PHE 160, GLU 193, LEU 197, ILE 212, LEU 231, VAL 163, LYS 178, PHE 228, LEU 230, ALA 176, GLU 229.	O sp^3 (O3) – N sp^3 from LYS 178<br>O sp^3 (O3) – O sp^2 from GLU 193<br>O sp^3 (O2) – O sp^2 from GLU 193<br>O sp^3 (O2) – N sp^3 from ASP 295	2.975<br>2.775<br>2.943<br>3.031
**Isolariciresinol**	-69.22	0.55	LEU 197, GLU 193, ILE 212, PHE 228, GLU 229, PHE 296, GLY 297, ASP 295, ILE 294, LYS 178, PHE 160, LEU 230, ALA 176, LEU 231, **SER** 232, VAL 163, LEU 282, ILE 155, MET 233, GLY 156, ASN 234, LYS 157, GLU 279, VAL 293, LEU 177.	O sp^2 (O2) – N sp^2 from LEU 231<br>O sp^3 (O2) – O sp^2 from GLU 229<br>O sp^3 (O3) – N sp^2 from ASP 295<br>O sp^3 (O5) – N sp^2 from ASN 234	3.195<br>3.188<br>3.108<br>3.107
**Lariciresinol**	-76.57	1.98	PHE 160, LYS 153, VAL 154, LYS 178, VAL 163, GLU 156, ILE 155, ALA 176, GLU 193, PHE 296, ASP 295, PHE 229, LEU 197, ILE 294, ASN 280, LEU 282, GLU 229, ILE 212, **SER** 232, LEU 282, LEU 231.	O sp^3 (O3) – N sp^2 from LYS 178<br>O sp^3 (O5) – O sp^2 from GLU 193<br>O sp^3 (O5) – N sp^2 from ASP 295	3.340<br>2.921<br>3.340
**Ligstroside-aglycone**	-76.01	0.13	LEU 197, ILE 212, PHE 228, GLU 229, LEU 230, LEU 231, ALA 176, **SER** 232, VAL 293, ILE 294, GLU 293, PHE 296, ASP 295, GLY 297, LYS 178, LEU 282, MET 233, VAL 163, ASN 234, ILE 155, GLY 156, GLU 279. GLU 237.	O sp^2 (O6) – N sp^2 from LEU 231<br>O sp^3 (O7) – O sp^2 from GLU 193<br>O sp^3 (O7) – N sp^2 from PHE 296<br>O sp^3 (O7) – N sp^2 from ASP 295	2.630<br>2.516<br>3.227<br>3.154
**Luteolin**	-71.01	0.01	PHE 160, GLY 297, GLU 193, PHE 296, ASP 295, LEU 197, ILE 294, ILE 212, LEU 282, PHE 228, ALA 176, **SER** 232, LEU 231, LEU 230, GLU 229, ILE 155, VAL 163, LYS 178	O sp^3 (O2) – O sp^2 from LEU 231<br>O sp^3 (O2) – N sp^2 from LEU 231<br>O sp^2 (O3) – N sp^2 from LEU 231<br>O sp^3 (O4) – O sp^2 from ILE 155<br>O sp^3 (O6) – N sp^2 from ASP 295<br>O sp^3 (O6) – O sp^2 from GLU 193	3.053<br>2.779<br>2.849<br>2.610<br>3.371<br>2.860
**Matairesinol**	-72.12	1.54	PHE 296, GLY 297, GLU 193, LEU 197, ASP 295, ILE 294, ILE 212, LEU 282, ASN 234, GLU 237, MET 233, **SER** 232, LEU 231, ILE 155, LEU 230, GLU 229, PHE 228, LYS 178, LEU 177, ALA 176, VAL 163, VAL 164.	O sp^3 (O4) – N sp^2 from ASP 295<br>O sp^3 (O6) – N sp^3 from LYS 178<br>O sp^3 (O6) – O sp^2 from GLU 193	2.648<br>3.284<br>3.052

**Medioresinol**	-72.31	0.02	LEU 197, PHE 296, GLU 193, GLY 297, ILE 212, ILE 294, ASP 295, GLU 229, PHE 228, LEU 230, ASN 280, LEU 282, LEU 231, GLU 279, ASN 234, MET 233, GLU 237, **SER** 232, ALA 176, LEU 177, LYS 178, PHE 160, VAL 163, VAL 164, ILE 155, GLY 156.	O sp^3 (O3) – N sp^2 from ASN 234<br>O sp^3 (O5) – N sp^3 from LYS 178<br>O sp^3 (O6) – N sp^2 from ASN 234 O<br>sp^3 (O7) – O sp^2 from GLU 193<br>O sp^3 (O7) – N sp^2 from PHE 296<br>O sp^3 (O7) – N sp^2 from ASP 295	2.716<br>3.239<br>3.068<br>3.448<br>3.091<br>2.830
**Oleacein**	-73.23	1.54	LYS 153, ALA 176, LYS 178, PHE 228, VAL 163, LEU 230, ILE 155, GLY 156, LEU 231, **SER** 232, MET 233, GLU 237, LEU 282, ASN 234, VAL 293, ILE 294, ASP 295, PHE 296, GLY 297, LEU 197, GLU 193, ILE 212.	O sp^2 (O3) – N sp^2 from ASN 234<br>O sp^2 (O3) – N sp^2 from ASN 234<br>O sp^3 (O5) – O sp^2 from GLU 193<br>O sp^3 (O5) – N sp^3 from LYS 178<br>O sp^3 (O7) – N sp^2 from ASP 295<br>O sp^3 (O7) – O sp^2 from GLU 193<br>O sp^3 (O7) – N sp^2 from PHE 296	3.028<br>2.910<br>2.803<br>3.044<br>3.068<br>2.752<br>3.223
**Oleocanthal**	-66.17	1.95	LEU 197, PHE 296, GLU 193, GLY 297, ILE 212, VAL 293, PHE 228, ILE 294, ASP 295, LYS 178, VAL 163, GLY 156, ILE 155, GLU 279, ASN 280, ALA 176, GLU 229, LEU 231, LEU 282, LEU 230, **SER** 232, MET 233, ASN 234	O sp^3 (O5) – O sp^2 from GLU 193<br>O sp^3 (O5) – N sp^3 from LYS 178	2.789<br>3.155
**Oleuropein aglycone**	-72.40	0.93	LEU 197, GLU 193, ILE 212, PHE 228, GLU 229, PHE 296, GLY 297, ASP 295, ILE 294, LYS 178, PHE 160, LEU 230, ALA 176, LEU 231, **SER** 232, VAL 163, LEU 282, ILE 155, MET 233, GLY 156, ASN 234, LYS 157, GLU 279, LEU 235, GLU 237	O sp^3 (O1) – N sp^2 from ASN 234<br>O sp^3 (O3) – N sp^2 from ASN234<br>O sp^3 (O3) – N sp^2 from ASN234<br>O sp^3 (O8) – O sp^2 from GLU 193<br>O sp^3 (O8) – N sp^3 from LYS 178	2.778<br>2.705<br>2.969<br>3.013<br>2.915
**Pinoresinol**	-79.04	0.04	GLU 193, GLU 297, LEU 197, PHE 296, ASP 295, PHE 160, LYS 178, VAL 163, ALA 176, PHE 228, ILE 155, GLU 229, LEU 230, ILE 294, ILE 212, ASN 286, LEU 231, **SER** 232, ASN 234, LEU 282, MET 233, GLU 237.	O sp^3 (O4) – N sp^3 from LYS 178<br>O sp^3 (O6) – N sp^2 from ASP 295<br>O sp^3 (O6) – N sp^2 from PHE 296<br>O sp^3 (O6) – O sp^2 from GLU 193	3.177<br>3.022<br>3.277<br>2.450
**Secoisolariciresinol**	-77.14	1.70	LEU 177, LYS 178, PHE 228, ALA 176, GLU 229, GLU 193, LEU 197, VAL 163, LEU 230, PHE 160, PHE 295, ILE 212, GLY 297, PHE 296, LEU 231, ASP 295, ILE 294, ILE 155, LYS 157, GLY 156, **SER** 232, MET 233, LEU 282, ASN 280.	O sp^3 (O3) – N sp^3 from LYS 178<br>O sp^3 (O5) – O sp^2 from GLU 193<br>O sp^3 (O5) – N sp^2 from ASP 295<br>O sp^3 (O5) – N sp^2 from PHE 296<br>O sp^3 (O6) – O sp^2 from LEU 231	3.377<br>2.926<br>2.674<br>3.332<br>2.982

**Syringaresinol**	-67.68	0.35	VAL 293, LEU 197, ILE 212, PHE 296, GLU 193, ILE 294, GLY 297, ASP 295, ASN 280, LEU 282, GLU 279, ASN 234, PHE 160, MET 233, GLU 237, GLY 156, ILE 155, LYS 165, VAL 163, **SER** 232, ALA 176, LYS 178, LEU 230, LEU 231, GLU 229, PHE 228.	O sp^3 (O3) – N sp^3 from LYS 178<br>O sp^3 (O7) – O sp^2 from GLU 193<br>O sp^3 (O7) – N sp^2 from ASP 295	3.088<br>2.704<br>3.228
**Vanillic acid**	-45.02	0.02	PHE 160, ASN 280, VAL 163, LEU 282, ALA 176, LEU 231, GLU 229, PHE 228, ILE 212, LEU 197, GLU 193, PHE 296, ILE 294, LYS 178, ASP 295.	O sp^3 (O2) – O sp^2 from GLU 193<br>O sp^3 (O2) – N sp^2 from ASP 295	2.756<br>3.118
**Verbascoside**	-88.14	1.09	LYS 153, VAL 154, LYS 165, ALA 176, VAL 163, LYS 178, GLY 156, PHE 228, LEU 230, GLU 229, LEU 231, **SER** 232, MET 233, GLU 193, GLY 297, LEU 197, ASP 295, PHE 296, ILE 294, GLU 279, ASN 280, ILE 212, LYS 240, LEU 282, ASN 234, GLU 237, MET 233, **SER** 232, LEU 231.	O sp^3 (O8) – O sp^2 from LEU 231<br>O sp^3 (O9) – O sp^3 from **SER** 232<br>O sp^3 (O9) – O sp^2 from MET 233<br>O sp^2 (O11) – N sp^2 from ASN 234<br>O sp^2 (O11) – N sp^2 from ASN 234 O<br>sp^3 (O13) – N sp^3 from LYS 178<br>O sp^3 (O13) – O sp^2 from GLU 193	3.090<br>3.339<br>3.134<br>2.857<br>2.912<br>3.185<br>2.897
**Tyrosol**	-46.56	0.11	ILE 212, GLU 229, PHE 228, ILE 294, PHE 296, GLU 193, GLU 297, ASP 295, LEU 282, LEU 231, LEU 230, ALA 176, VAL 163, LYS 178, GLU 229.	O sp^3 (O1) – O sp^2 from GLU 229<br>O sp^3 (O2) – O sp^2 from GLU 193	3.040<br>2.957

Table S2. The list of the intermolecular interactions between the native ligand (curcumin) and the 20 test vegetal compounds docked with 5ZTN (MVD Software)

Test Compounds	MolDock Score	Molecule Contributions	Hydrogen bond	Bond Length Å	Steric Interactions	Distance Å
**Co-crystallized CUR A501 CURCUMIN (native ligand)**	-115.85	ALA 176, ASP 295, GLU 193, GLU 229, GLY 297, ILE 155, ILE 212, ILE 294, LEU 197, LEU 230, LEU 231, LEU 282, LYS 153, LYS 165, LYS 178, MET 233, PHE 160, PHE 228, PHE 296, SER 232, VAL 163	O sp^2(O1) - N $sp^3$from LYS 178<br>O sp^3 (O3) - N sp^3 from LYS 178<br>O sp^3 (O3) - O sp^3 from GLU 193	3.102<br>2.661<br>2.249	C sp^2(C22)- N $sp^2$from GLU 193<br>C sp^2(C18)- N $sp^2$from GLU 193<br>O sp^3 (O3) – C sp^2 from GLU 193<br>O sp^2 (O3) – C sp^3 from LYS 178<br>C sp^3(C15) - O $sp^2$from LEU 231<br>C sp^3(C23) - N $sp^2$from LEU 231	3.12<br>3.05<br>3.03<br>3.13<br>2.80<br>2.74
**ANEMONIN**	-93.75	ALA 176, ASP 295, GLU 193, GLU 229, ILE 212, ILE 294, LEU 177, LEU 197, LEU 231, LEU 282, LYS 178, PHE 160, PHE 228, PHE 296, VAL 163, VAL 293	O sp^3 (O1) – N sp^2 from ASP 295	2.846	C sp^2 (C13) – C sp^2 from ASP 295<br>O sp^3 (O1) – C sp^3 from ILE 294<br>C sp^3 (C7) – C sp^3 from ILE 294<br>C sp^2 (C12) – C $sp^3$from ALA 176	3.04<br>3.15<br>3.17<br>3.14
Apigenin	-101.31	ALA 176, ASP 295, GLU 193, GLU 229, ILE 155, ILE 212, ILE 294, LEU 197, LEU 230, LEU 231, LEU 282, LYS 178, PHE 228, PHE 296, VAL 163	O sp^3 (O2) – O sp^3 from GLU 193	2.917	O sp^2 (O3) – C sp^3 from ILE 212<br>O sp^2 (O3) – C sp^3 from ILE 212<br>C sp^2 (C10) – N $sp^2$from LEU 231	3.09<br>2.95<br>3.02

o-Coumaric acid	-71.56	ALA 176, ASP 295, GLU 193, GLU 229, ILE 155, ILE 212, ILE 294, LEU 197, LEU 230, LEU 231, LEU 282, LYS 178, PHE 160, PHE 228, PHE 296, SER 232, VAL 163	O sp^2(O3) - N $sp^3$from LYS 178<br>O sp^3 (O2) – O sp^3 from GLU 193<br>O sp^3 (O2) – O sp^3 from PHE 296	3.500<br>2.855<br>3.563	O sp^2 (O3) – N sp^2 from ASP 295	3.10
Elenoic acid	-68.71	ALA 176, ASN 234, GLU 229, GLU 279, GLY 156, ILE 155, ILE 212, ILE 294, LEU 230, LEU 231, LEU 282, LYS 165, LYS 178, MET 233, PHE 160, PHE 228, SER 232, VAL 163	O sp^2 (O2) – N $sp^2$from ASN 234<br>O sp^2 (O2) – N $sp^2$from ASN 234<br>O sp^3 (O4) – N sp^2 from ILE 155	3.139<br>3.302<br>3.006	C sp^2 (C16) – N $sp^2$from LEU 282	3,24
Hydroxytyrosol	-73.62	ALA 176, ASP 295, GLU 193, GLU 229, ILE 212, ILE 294, LEU 197, LEU 230, LEU 231, LEU 282, LYS 178, PHE 228, PHE 296, VAL 163	O sp^3(O3) - N $sp^3$from LYS 178<br>O sp^3 (O3) – O sp^3 from GLU 193<br>O sp^3 (O2) – O sp^3 from GLU 193<br>O sp^3 (O1) – O sp^2 from GLU 229	3.117<br>2.591<br>3.531<br>2.957	O sp^3 (O2) – N sp^2 from ASP 295<br>O sp^3(O3) - C $sp^3$from LYS 178	2.93<br>3.15
Isolariciresinol	-114.37	ALA 176, ASN 234, ASN 280, ASP 295, GLU 193, GLU 229, GLU 279, GLY 156, ILE 155, ILE 212, ILE 294, LEU 197, LEU 230, LEU 231, LEU 282,	O sp^3(O3) - N $sp^3$from ASP 295<br>O sp^3 (O4) – N $sp^3$from LYS 178<br>O sp^3 (O2) – O sp^2 from ILE 155<br>O sp^3 (O1) – N sp^2 from LEU 231	3.151<br>3.159<br>3.140<br>3.336	C sp^3 (C25) – C sp^3 from ILE 212<br>O sp^3 (O5) – O sp^2 from GLU 279<br>C sp^3 (C26) – N sp^2 from ASN 234<br>C sp^2 (C22) – C $sp^3$from ILE 294	2.56<br>3.11<br>3.12<br>3.86
		LYS 157, LYS 178, PHE 160, PHE 228, PHE 296, VAL 163	O sp^3 (O1) – O sp^2 from GLU 229	2.698	C sp^2 (C23) – C $sp^2$from PHE 160<br>C sp^2 (C19) – C $sp^3$from VAL 193	3.06<br>2.64
Lariciresinol	-127.68	ALA 176, ASN 234, ASP 295, GLU 193, GLU 229, GLU 279, GLY 156, ILE 155, ILE 212, ILE 294, LEU 197, LEU 230, LEU 231, LEU 282, LYS 178, MET 233, PHE 228, PHE 296, SER 232, VAL 163, VAL 293	O sp^3(O2) - N $sp^2$from LEU 231<br>O sp^3 (O4) – O $sp^2$from ASP 295<br>O sp^3 (O6) – O sp^3 from GLU 193<br>O sp^3 (O6) – N sp^3 from LYS 178	3.068<br>2.660<br>2.880<br>3.219	C sp^3 (C12) – C sp^3 from ASP 176<br>C sp^3(C26)- N $sp^2$from GLU 193<br>O sp^3(O6) - C $sp^3$from LYS 178	3.12<br>2.87<br>2.96
Ligstroside-aglycone	-102.50	ALA 176, ASN 234, ASN 280, ASP 295, GLU 229, GLU 279, GLY 156, GLY 158, ILE 155, ILE 212, ILE 294, LEU 230, LEU 231, LEU 282, LYS 157, LYS 277, MET 233, PHE 160, PHE 228, PHE 296, SER 232, VAL 163	O sp^3(O7) - N $sp^3$from LYS 277<br>O sp^2 (O2) – N sp^2 from LEU 231	2.878<br>2.618	O sp^2 (O2) – O sp^2 from GLU 229<br>C sp^3(C19)- N $sp^2$from GLY156	3.11<br>3.14

Luteolin	-104.91	ALA 176, ASP 295, GLU 193, GLU 229, GLY 156, ILE 155, ILE 212, ILE 294, LEU 230, LEU 231, LEU 282, LYS 178, PHE 228, PHE 296, SER 232, VAL 163	O sp^2 (O2) – N sp^2 from LEU 231<br>O sp^3 (O1) – O sp^3 from LEU 231<br>O sp^3 (O3) – O sp^2 from ILE 155<br>O sp^3 (O5) – O sp^3 from GLU 193	2.814<br>2.818<br>2.573<br>2.775	O sp^2 (O2) – C sp^2 from LEU 231<br>O sp^2 (O2) – O sp^2 from GLU 229	3.15<br>2.70
Matairesinol	-116.98	ALA 176, ASN 234, ASP 295, GLU 193, ILE 155, ILE 212, ILE 294, LEU 230, LEU 231, LEU 282, LYS 178, MET 233, PHE 160, PHE 228, PHE 296, SER 232, VAL 163	O sp^3 (O6) – O sp^3 from GLU 193<br>O sp^3 (O6) – N sp^3 from LYS 178	2.760<br>3.077	O sp^3 (O6) – C sp^3 from LYS 178<br>O sp^2 (O2) – C sp^3 from PHE 228<br>C sp^2 (C14) – C sp^2 from PHE 228<br>C sp^3 (C25) – C sp^3 from ILE 294<br>C sp^3 (C11) – C sp^3 from ILE 294<br>C sp^3 (C9) – C sp^{3}from LEU 282<br>C sp^3 (C7) – C sp^{3}from LEU 282	3.01<br>2.32<br>2.94<br>3.18<br>2.79<br>3.08<br>3.14
Medioresinol	-124.68	ALA 176, ASN 234, ASN 280, ASP 295, GLU 193, GLU 229, GLU 237, GLU 279, GLY 156, ILE 155, ILE 212, ILE 294, LEU 197, LEU 230, LEU 231, LEU 282, LYS 178, MET 233, PHE 160, PHE 228, PHE 296, SER 232, VAL 163	O sp^3(O6) - N sp^{2}from ASN 294<br>O sp^3 (O4) – N sp^{2}from ASN 294<br>O sp^3 (O7) – N sp^2 from PHE 296<br>O sp^3 (O7) – O sp^3 from GLU 193<br>O sp^3 (O5) – N sp^3 from LYS 178	2.991<br>2.627<br>3.133<br>2.752<br>3.533	C sp^3 (C10) – C sp^{3}from LEU 282<br>C sp^3 (C12) – C sp^{3}from VAL 163<br>C sp^3 (C12) – C sp^{3}from ALA 176<br>C sp^2 (C25) – N sp^2 from ASP 295<br>O sp^3 (O7) – C sp^3 from ASP 295	3.12<br>2.81<br>3.10<br>3.09<br>2.61
Oleacein	-88.52	ALA 176, ASN 234, ASP 295, GLU 193, GLU 229, GLU 237, GLY 156, ILE 155, ILE 212, ILE 294, LEU 282, LYS 178, MET 233, PHE 160, PHE 228, PHE 296, SER 232, VAL 163	O sp^3 (O6) – N sp^3 from LYS 178	3.159	C sp^3 (C13) – O sp^2 from ILE 155	3.06
Oleocanthal	-93.41	ALA 176, ASN 280, ASP 295, GLN 162, GLU 193, GLU 229, GLY 156, ILE 155, ILE 212, ILE 294, LEU 177, LEU 230, LEU 231, LEU 282, LYS 178, PHE 160, PHE 228, SER 232, VAL 163	O sp^3 (O5) – O sp^2 from ILE 155<br>O sp^2(O3) - N sp^3 from LYS 178<br>O sp^2(O4) - N sp^2 from ASP 295	3.073<br>3.308<br>2.759	O sp^3(O1) - C sp^3 from LEU 282<br>C sp^3(C17) - C sp^3 from LYS 178<br>C sp^3(C17) - C sp^3 from LYS 178<br>C sp^3(C17) - C sp^3 from LYS 178<br>C sp^3 (C17) – C sp^3 from VAL 163	3.20<br>2.90<br>3.15<br>2.75<br>2.42
Oleuropein aglycone	-88.49	ALA 176, ASN 234, GLU 229, GLU 279, GLY 156, ILE 155, ILE 212, ILE 294, LEU 230, LEU 231, LEU 282, LYS 153, LYS 165, LYS 178, MET 233, PHE 228, PHE 296, SER 232, VAL 163	O sp^3(O7) - N sp^3 from LYS 165<br>O sp^3 (O8) – N sp^3 from LYS 165<br>O sp^3 (O8) – N sp^3 from LYS 153<br>O sp^2 (O5) – O sp^3 from LEU 231	3.331<br>2.715<br>2.590<br>3.266	C sp^3 (C19) – C sp^3 from ILE 212	2.99

Pinoresinol	-126.85	ALA 176, ASP 295, GLU 193, GLU 229, GLY 156, GLY 297, ILE 155, ILE 212, ILE 294, LEU 197, LEU 230, LEU 231, LEU 282, LYS 178, MET 233, PHE 160, PHE 228, PHE 296, SER 232, VAL 163	O sp^3(O4) - N sp^3 from ASP 295<br>O sp^3(O4) - O sp^3 from ASP 295<br>O sp^3 (O6) – O sp^3 from GLU 193<br>O sp^3 (O6) – N sp^3 from LYS 178<br>O sp^3 (O5) – O sp^2 from LEU 231	3.142<br>2.909<br>2.955<br>2.698<br>3.137	O sp^3(O4) - O sp^3 from GLU 193<br>C sp^3(C24) - C sp^3 from ILE 212<br>O sp^3 (O6) – C sp^3 from ASP 295<br><br>(strong electrostatic interactions)	2.88<br>2.85<br>2.93
Secoisolariciresinol	-112.96	ALA 176, ASN 234, ASP 295, GLU 193, GLU 229, GLU 237, GLU 279, GLY 156, ILE 155, ILE 212, ILE 294, LEU 197, LEU 230, LEU 231, LEU 282, LYS 178, MET 233, PHE 160, PHE 228, PHE 296, SER 232, VAL 163	O sp^3(O4) - N sp^2 from ASN 234<br>O sp^3(O4) - N sp^2 from ASN 234<br>O sp^3(O6) - N sp^2 from ASN 234<br>O sp^3 (O6) – O sp^2 from GLU 279<br>O sp^3 (O2) – O sp^2 from GLU 229<br>O sp^3 (O2) – N sp^2 from LEU 231<br>O sp^3 (O3) – N sp^3 from LYS 178<br>O sp^3 (O5) – O sp^3 from GLU 193	3.393<br>3.010<br>2.642<br>3.191<br>3.329<br>2.974<br>3.206<br>2,716	C sp^3 (C12) – C $sp^3$from ALA 176<br>O sp^3 (O1) – C $sp^3$from LEU 282	3.10<br>2.74
Syringaresinol	-108.62	ALA 176, ASN 234, ASN 280, ASP 295, GLU 193, GLU 237, GLU 279, GLY 156, ILE 155, ILE 212, ILE 294, LEU 230, LEU 231, LEU 282, LYS 165, LYS 178, MET 233, PHE 160, PHE 228, PHE 296, SER 232, VAL 163	O sp^3 (O7) – N sp^3 from LYS 178	3.349	C sp^2 (C15) – C $sp^3$from ILE 294<br>C sp^2 (C19) – C $sp^3$from ILE 294	2.94<br>2.94
Vanillic acid	-69.88	ALA 176, ASP 295, GLU 193, GLU 229, ILE 212, ILE 294, LEU 197, LEU 282, LYS 178, PHE 160, PHE 228, PHE 296, VAL 163	O sp^3 (O2) – N sp^3 from LYS 178<br>O sp^3 (O1) – N sp^3 from ASP 295	2.973<br>3.069	O sp^3 (O1) – O sp^3 from GLU 193	3.03
Verbascoside	-148.78	ALA 176, ASN 234, ASN 280, ASP 295, GLN 286, GLU 193, GLU 237, GLU 279, GLY 156, ILE 155, ILE 212, ILE 294, LEU 197, LEU 230, LEU 231,	O sp^3 (O15) – N sp^3 from LYS 178<br>O sp^3 (O15) – O sp^3 from GLU 193<br>O sp^3(O1) - N sp^2 from ASN 234	3.080<br>2.548<br>3.365<br>3.397	C sp^2 (C37) – C $sp^3$from MET 233<br>O sp^3 (O8) – C sp^3 from GLU 279<br>C sp^3 (C23) – O sp^2 from GLU 279<br>O sp^3 (O11) – O sp^2 from ILE 155	3.19<br>3.07<br>3.03<br>2.58

		LEU 282, LYS 157, LYS 178, LYS 277, MET 233, PHE 160, PHE 228, PHE 296, SER 232, VAL 163	O sp^3 (O9) – O $sp^2$from ASN 280<br>O sp^3 (O9) – O sp^3 from ASP 295<br>O sp^3 (O8) – O $sp^2$from ASN 280<br>O sp^3 (O8) – O sp^2 from GLU 279<br>O sp^3 (O6) – O sp^2 from ILE 155<br>O sp^3 (O12) – O sp^3 from SER 232<br>O sp^3 (O13) – O sp^3 from SER232	3.515<br>2.589<br>3.097<br>3.540<br>3.105<br>2.985		
Tyrosol	-68.27	ALA 176, ASP 295, GLU 193, GLU 229, ILE 212, ILE 294, LEU 197, LEU 230, LEU 231, LEU 282, LYS 178, LYS 277, PHE 228, PHE 296, VAL 163	O sp^3 (O1) – O sp^2 from GLU 229<br>O sp^3 (O2) – O sp^3 from GLU 193	3.081<br>2.591	O sp^3 (O2) – N sp^2 from ASP 295	3.32

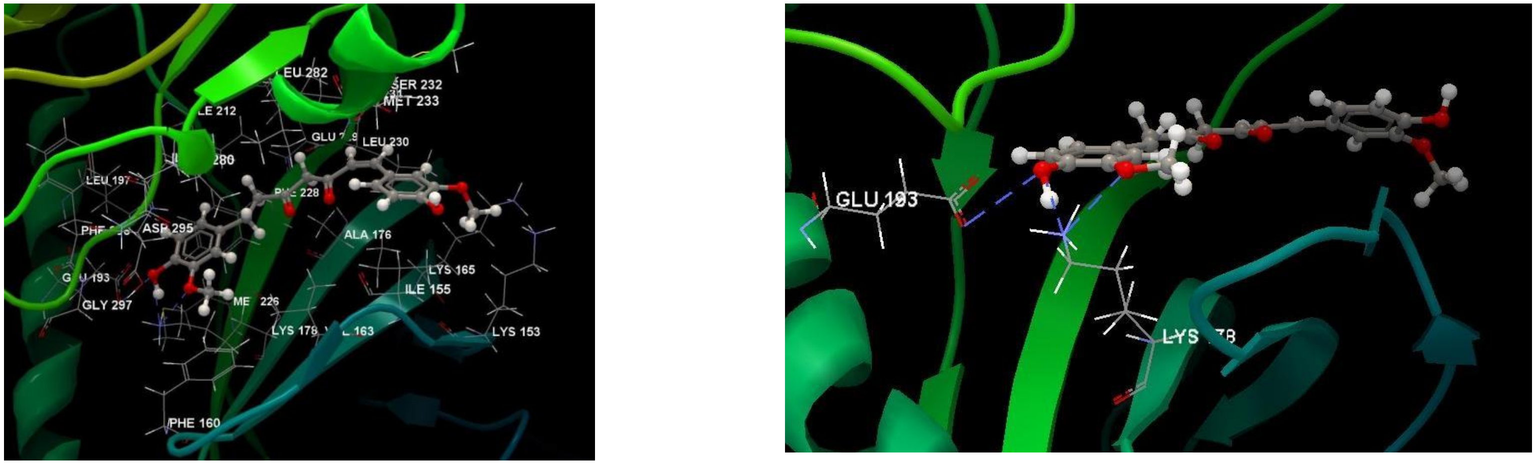

(a) (b)

Figure S1 (a and b). **CLC—**The images of the co-crystallized curcumin (the native ligand) interacting with the amino acid residues in the binding site of 5ZTN: (a) the general docking pose and (b) the hydrogen bonds between the native ligand and the active amino acids in the binding site, GLU 193 and YS 178.

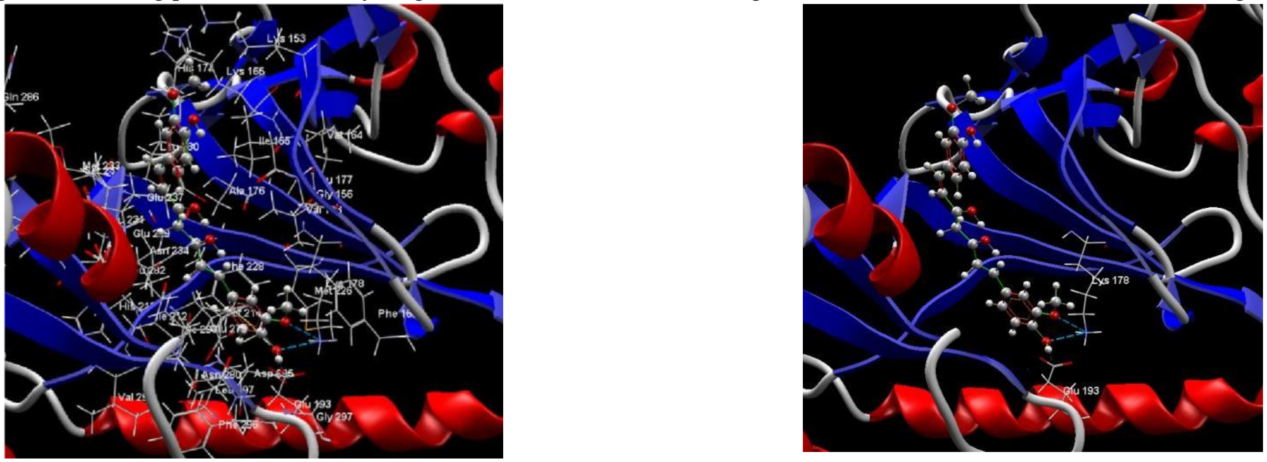

(a) **(b)**

Figure S2 (a and b). **MVD—**The images of the co-crystallized curcumin (the native ligand) interacting with the amino acid residues in the binding site of 5ZTN: (a) the general docking pose and (b) the hydrogen bonds between the native ligand and the active amino acids in the binding site, GLU 193 and YS 178.

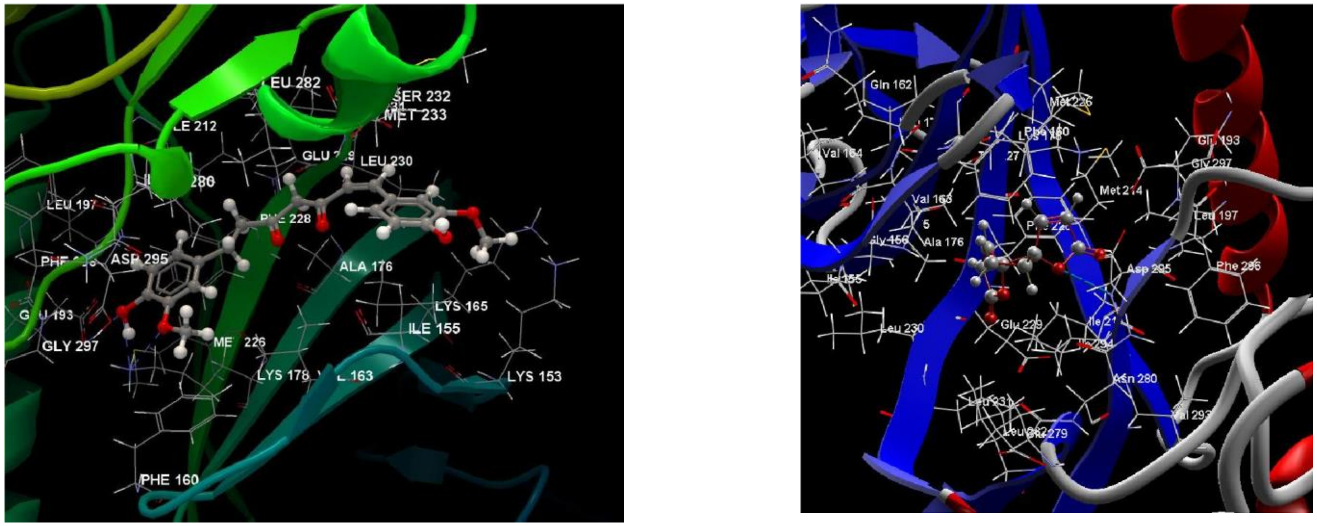

(a) **(b)**

Figure S3 (a and b). The images of anemonin interacting with the amino acid residues in the binding site of 5ZTN: CLC docking pose and (b) MVD docking pose.

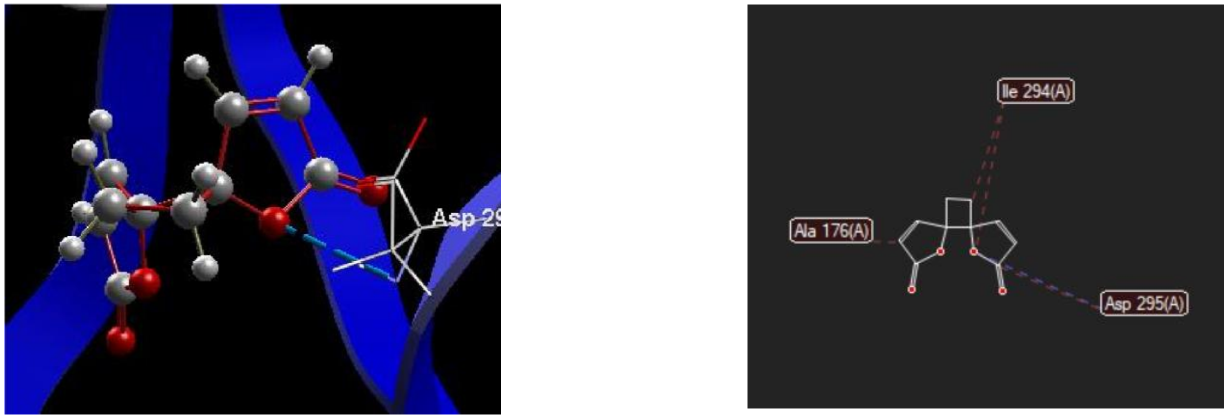

(**a**) (**b**)

Figure S4 (a and b). The images of anemonin interacting with the amino acid residues in the binding site of 5ZTN: (a) the hydrogen bonds with ASP 295; (b) hydrogen bonds (blue) steric interactions (red) between anemonin and the amino acids residues of the binding site of 5ZTN in 2D.
